# Who and how many can work from home? Evidence from task descriptions

**DOI:** 10.1186/s12651-021-00287-z

**Published:** 2021-02-28

**Authors:** Henning Holgersen, Zhiyang Jia, Simen Svenkerud

**Affiliations:** Statistics Norway, Post Box 2633, 0131 Oslo, Norway

**Keywords:** COVID-19, Working from home, Job advertisements, Unconventional data, D24, J22, J61, O30, R12, R32

## Abstract

The Covid-19 crisis has forced great societal changes, including forcing many to work from home (WFH) in an effort to limit the spread of the disease. The ability to work from home has long been considered a perk, but we have few estimates of how many jobs are actually possible to be performed from home. This paper proposes a method to estimate the share of these jobs. For each occupation, we obtain a WFH friendly measure by asking respondents from Amazon Mechanical Turk (MTurk) to evaluate whether the corresponding tasks can be performed from home based on the descriptions from the International Standard Classification of Occupations 2008 (ISCO-08) standard. The share of WFH friendly jobs in an economy can then be estimated by combining these measures with the labor statistics on occupational employments. Using Norway as an illustrating example, we find that approximately 38% of Norwegian jobs can be performed from home. The Norwegian results also suggest that the pandemic and the government’s attempts to mitigate this crisis may have a quite uneven impact on the working population. Those who are already disadvantaged are often less likely to have jobs that can be performed from home.

## Introduction

Covid-19 pandemic hit the world hard and unprepared. In a study of the Spanish flu, Hatchett et al.(2007) show that non-pharmaceutical interventions known as “social distancing” during a pandemic can significantly reduce the disease transmission and lower both the peak and cumulative excess mortality. Learning from the historical lessons, many countries implemented measures to limit physical contact between people. Encouraging working from home is an important part of such measures. Not all jobs can be performed away from offices. Workers with non-WFH friendly jobs^1^ will be hit harder by such policies, since they are forced into a situation where they have to choose between two unfortunate options: increased risk of infection or substantial economic loss due to lost work opportunities. Similarly, firms and regions with few WFH workers may be more severely impacted than others. Thus, the potential impact of “social distancing” policy and the Covid-19 crisis could be highly unequal. Identifying non-WFH workers would be essential for designing effective economic austerity packages. However, there is rather limited knowledge of the prevalence and characteristics of non-WFH workers.

In this paper, we propose a method to answer the question: who and how many can work from home in an economy. For this purpose, we evaluate the WFH feasibility for the 426 occupations listed in the ISCO-08 (International Labor Organization 2012). In particular, respondents from an on-line labor marketplace, Amazon Mechanical Turk (MTurk) (Amazon 2020), are asked to evaluate whether they think occupations can likely be performed from home using the detailed descriptions of tasks to be performed. Based on the responses, we establish a WFH friendly measure for each occupation. Combing these measures with occupational employment statistics, we can obtain information on the prevalence of jobs that can be worked from home. If in addition employment information on individual level is available, we can link workers, jobs and occupations together and identify what type of workers are less likely to have WFH friendly jobs. This method can be easily applied to other economies of interest. In current paper, we focus on Norway and use it as illustrative example. The patterns we find based on Norwegian data may be informative for other industrial countries since the occupational structures in these economies are often similar.

We find that approximately 38% of Norwegian jobs can be performed at home. WFH-friendly jobs typically pay better than non-WFH friendly jobs. The prevalence of such jobs varies a lot across geographical areas. There is a larger share of WFH friendly jobs in urban than in rural areas. More importantly, as many worried, workers who are already disadvantaged in the labor market, such as young workers, workers with migrant background, low educated and lone parents, are often less likely to have WFH friendly jobs. We have also combined our WFH friendly measures with country specific employment data from Eurostat and estimated the prevalence of WFH friendly jobs in other European countries. We find that rich and more developed countries have larger shares of jobs that can be performed from home than poor and less developed ones.

The rapid development of information and communication technologies (ICTs) has revolutionized the way that work is organized. Many workers can now perform their tasks and connect with their colleagues from any place. The implications of this new way to organize work have been studied extensively. Among them, many studies try to estimate the share of workers that *actually* work from home. They find that there are relative large differences across different countries, sectors and firms. See for example, studies by Gschwind and Vargas (2019), Lister and Harnish (2019) for an review. Almost all these studies, including the most recent ones (Alipour et al. 2020; Barbieri et al. 2020; Mongey et al. 2020), are based on existing surveys. One exception is a study using US data by Dingel and Neiman (2020). While their main analysis is based on the Occupational Information Network (O*net) surveys with questions covering “work context” and “generalized work activities, they have also tried to manually evaluate WFH feasibility for each occupation themselves. Existing surveys are often designed for other purposes so that information obtained may not directly answer our question of interest. What we are interested in this paper is whether a given job can be *potentially* worked from home, which in principal depends on only the nature of tasks that need to be performed. These surveys typically provide information on actual incidences of home office, which in addition depend on many contextual factors, such as regulations, working cultures, attitudes of workers and managers and etc. Moreover, the surveys are often implemented some years back which may lead to concerns of timeliness. Considering the rapid technological progress in information technology, this concern may be particularly relevant. On the other hand, new surveys on a representative sample of the working population are costly in term of both time and resource, which makes it not a practical alternative for our purpose. The method we used in this paper is similar to the manual evaluation method applied by Dingel and Neiman (2020). However, we don’t do evaluations ourselves but rely on respondents from MTurk. While we acknowledge that well-designed surveys are still the most reliable source, our study suggests an unconventional data source where reliable information can be easily obtained timely with much less cost.

There are clear limitations with our method. For example, the respondents from MTurk are most likely not expert in the field of interest. Or the description of tasks may not be entirely clear. These issues will lead to potential bias in our measure. Although we cannot directly test the reliability of our WFH friendly measures, we have done several consistency checks and we did not find evidence of large bias. We first compare our results with that of Dingel and Neiman (2020). We find that these two measures are very similar on the ISCO-08 major group occupation level (correlation 0.96), although they differ somewhat on the unit group occupation level (correlation 0.65). Dingel and Neiman (2020)’s results also predict a higher share of jobs which can be done from home than ours, but the difference is small (43% vs 38%). In the second attempt to check the consistency of our measures, we compare our results with observed WFH incidence from previous surveys in Norway. There have been two surveys in Norway that include questions on whether the respondents actually work from home: The Norwegian labor force survey in 2017 and a recent survey by the Norwegian Institute of Transport Economics (TØI). Although the actual WFH incidence is not the same as the potential capacity of WFH, their results and ours are are broadly similar. Finally, we use Norwegian job advertisements data published by the Norwegian welfare administration (NAV) between January 2012 and March 2019. Some of these advertisements mentioned possibilities of WFH to attract more candidates. We identify those advertisements and construct the relative frequencies of remote-friendly jobs across 9 major ISCO-08 occupational groups. A comparisons between the observed frequencies and those predicted using our results could be a crude way for quality check. We find that the NAV job ads data provide supports to our WFH friendly measures. While none of these checks can directly prove the reliability of our measure, they do suggest that our measures are consistent with several observable empirical patterns and help relieve the concern on the quality of our measure.

## Background

Even before the Covid-19 pandemic, there is already an increasing number of workers who regularly perform their work away from the office. This practice is often considered as a potential solution to many social and organizational problems (Bailey and Kurland 2002). This new work mode represents a fundamental change that has substantial impacts on workers, employers and society. Since the early contribution by Nilles (1975), a large body of literature has been developed. However, the discussions remain to be intense despite of increasing understanding of this new work mode. Big companies such as Yahoo, Best Buy and HP ended their work from home programs in 2010s due to worries on possible negative impacts on performance. Hubert Joly, Best Buy CEO at the time, stated that Best Buy’s working from home program was “fundamentally flawed from a leadership standpoint” (Allen et al. 2015). Many criticize that these companies make these programs the scapegoat for their bad managements. This criticism might be unjustified, since there is no consensus in the literature on how work from home influence the worker’s productivity. The effect of WFH on productivity is theoretically ambiguous, while empirical evidences are mixed. Bloom et al. (2014) suggest that WFH increase the productivity of workers based on a randomized controlled trial in a large Chinese travel agency. Monteiro et al. (2019) find the opposite effects using a large panel of Portuguese firms. Glenn Dutcher (2012) suggests that the effect of WFH may be heterogeneous: it may have positive implications on productivity of creative tasks but negative implications on productivity of dull tasks. A complete review of researches on this new work mode is beyond of the scope. Interested readers are refer to those by Messenger (2019), Allen et al. (2015), Siha and Monroe (2006) and Bailey and Kurland (2002).

There are several closely related and often confused concepts in the literature which we need to distinguish: “Telework”, “Remote work”, “Mobile work” and “Working from home”. Originating from Nilles (1975), “Telework” refer to work arrangements where tasks are performed away from the employer’s premises with the help of ICTs (Messenger 2019). International Labor Organization (2020) consider “Telework” as a subcategory of the broader concept “Remote work”. They claim that “What makes telework a unique category is that the work carried out remotely includes the use of personal electronic devices”. However, there are rather few jobs that can be done remotely without requiring the use of ICTs nowadays, which makes this distinction less useful. In practice, the use of “Remote work” is often intended to stress the geographically detachment between work and fixed places of work. U.S. Office of Personnel Management (2013) goes even further and considers only worker who “resides and works at a location beyond the local commuting area of the employing organization’s worksite” as “Remote worker”. On the other hand, “Working from home” emphasizes that it is the location (worker’s own home) in which the work is performed. It rules out any non-home-based forms of “Remote work”. Traditionally, “Mobile work” is associated with work arrangements that require workers to spend most of their time out of the office (Siha and Monroe 2006), such as door to door salesman. More recently, the terminology “Mobile work” or “Mobile office” is used to highlight the fact that work could be done not only at office or home, but various locations in between, such as cafes, hotels, airport lounges and etc.

In this paper, our goal is to evaluate the workers’ ability to continue to work while avoiding or minimizing physical contacts with others. Among the terms we discussed above, “Working from home” is probably the best suited definition for this purpose. There is a relative large literature that tries to measure how many actually work away from the office. However, the estimates often vary considerably. These estimates involve typically different definitions (as discussed above), have different reference populations (all workers or only a particular group of workers) and are based on different thresholds on the intensity of home working (occasionally or regularly). More importantly, these estimates measure the actual observed incidences. Even if the tasks in principal can be performed from home, whether a worker actually work from home depends on many different factors. For example, employment regulations on working time and workplace flexibility can play important roles (Gschwind and Vargas 2019). Working culture, such as management’s trust of workers, is another important factor (Bailey and Kurland 2002). While this strand of literature is highly relevant, it cannot directly answer our question of interest: what jobs are technically possible to be performed at home.

A note on nomenclature: For brevity, we sometimes refer to “WFH friendly” occupations rather than” occupations of which required tasks can be performed from home”. We use the terms interchangeably. This does not mean that such employees in actuality work from home either permanently or occasionally.

## Method

As mentioned above, the incidence estimates from previous studies do not directly address our question of interest. A traditional survey that asks workers whether they consider their job tasks can be performed from home or not could provide the most reliable answer to our question. However this can be quite costly, in terms of both time and resources. In this paper, we propose a simple, cheap and timely method by noting that different jobs often have similar tasks and duties undertaken and can be organized into a limited number of occupations. We evaluate, not for every job but every occupation, the feasibility of WFH. While this greatly reduces the workload needed, it ignores possible heterogeneity across jobs in the same occupation and may introduce bias, which we discuss in more detail below.

We group the jobs into occupations following the ISCO-08 standard. The ISCO-08 contains 9 major occupation groups and 426 occupations (Armed forces occupations are excluded in this analysis) at the unit group level. Detailed task descriptions for these occupations are listed in the ISCO-08 documentation. Using these descriptions, we try to evaluate whether an occupation is likely to be performed from home. To do this, we make use the online platform MTurk, which is “a marketplace for work that requires human intelligence” (Amazon 2020). Users of MTurk can generate different tasks that MTurk workers work on. It has gained increasing popularity in social sciences. Researches have shown that MTurk can provide quick and reliable responses at relatively low costs (Buhrmester et al. 2011; Berinsky et al. 2012).

In order to increase the quality of our WFH friendly measure in a resource-effective manner, we adopt a system of evaluation loosely inspired by the Delphi method (Ziglio and Adler 1996), adopted for MTurk. The Delphi method was originally created for situations where researchers were unsure of what questions to ask for a survey, and outlined a process by which a group of experts were consulted iteratively in order to reach a consensus and find possible areas of contention. The differences to MTurk may seem stark—MTurk workers are not experts, nor can we consult the same MTurk workers repeatedly. Instead, we can view the occupations with a consensus as non-contentious issues, where the panel (MTurk workers) largely understood the question and agreed on the answer. The remainder are issues where either the question (occupation description) was misformed, there was real disagreement, or where there was excessive noise in the replies. We do not return not to the same group of respondents but to a more reliable group with more informative questions, and we can get more MTurk workers to answer each question. Research (Peer et al. 2014) also indicates a large quality improvement from by requiring MTurk workers to have had an high acceptance rate from previous work. However, we realize that while making these changes will help to potentially improve the reliability of our answers. They did not resolve possible systematic biases that caused by the fact that MTurk workers are not a representative sample of the working population.^2^

For our purpose, we create labelling jobs for each occupation. The labelling jobs are done in two consecutive rounds. In the first round, all 426 occupations are presented to at least 5 MTurk workers for classification. Only those occupations on which the MTurk workers largely disagree (less than 80% MTurk workers agree) were included in the second round.^3^ In the following we will label these occupations as the occupations that lack of consensus. In this round, we make three changes in contrast to round one. First, we increase the number of responses to 15, so that totally we have at least 20 answers for each of these occupations. Second, we refine and expand the descriptions of tasks in more detail. Finally, only workers with high acceptance rates are allowed to participate in the labelling job. We hope this could help us to further improve the quality of the classification.

### The first round

In the first round, each occupation was presented together with a brief description. The exact question formulation was “Can this type of job likely be performed from a home office?”, and an example of a job description could be:

*Electrical engineers conduct research and advise on, design, and direct the construction and operation of electrical systems, components, motors and equipment, and advise on and direct their functioning, maintenance and repair, or study and advise on technological aspects of electrical engineering materials, products and processes.*

The respondent was asked to evaluate whether it was likely that the job could be performed primarily from a private home. The alternatives were “Yes”, “No” and “Unknown”, which were provided with the following description:

1. *Yes: This job can be performed primarily from an office in a private home*
2. *No: Substantial parts of this job must be performed outside the employees home*
3. *Unknown: There is not enough information to decide.*

**Fig. 1:**
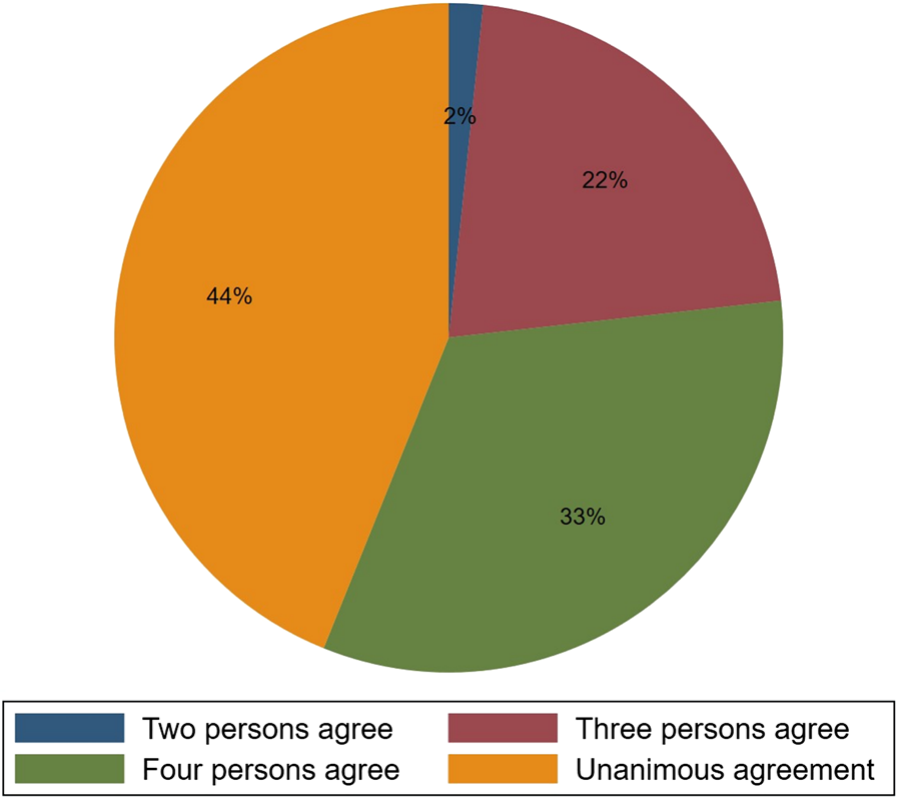
Agreement among the respondents from MTurk

We provided an “unknown” option in addition to the “yes/no” options in order to reduce arbitrary responses to uninformative occupation descriptions. In order to reduce the serendipity in the labels, we acquired at least five labels from different respondents independently for each occupation. As expected, these respondents are not always agree with each other. Figure 1 shows how they agree/disagree. For the majority of occupations (around 77% of occupations), there are at least 4 respondents agree on the same answer. Interestingly, the “unknown” label is seldom used: it is assigned 40 times, less than 2% of all labels assigned. Thus, the disagreement among respondents is probably not an indication that some occupations are difficult to evaluate for the respondents. We conclude that the occupations with a consensus of at least 4 are sufficiently certain that we can accept the answer, while the remaining 23–24% are needs to be redone.

There may be several reasons for this lack of consensus, one welcome and two unwelcome:

- There are real differences in opinion as to whether a job can be done from home.
- Turks are opportunistic, answering at random to minimize time spent on each task.
- The information provided was insufficient, leaving workers to fill in the blanks themselves.

Among these three factors, real differences in judgement may be informative and increase our measure accuracy by incorporating several experiences and views on remote feasibility. The two other reasons for lack of consensus can be combated by collecting more labels from”more responsible” MTurk workers and by providing a better description of a job.

### The second round

The occupations with a clear lack of consensus are annotated twice more, using two different MTurk panels. One panel of 10 workers who all are “masters” according to MTurk (a simple albeit vague checkbox indicating a high acceptance rate), and a panel of 5 workers who, in addition to being “masters”, have an task acceptance rate above 80% (meaning that at least 80% of their work has been accepted by other MTurk users). The threshold of 80% was chosen mostly at random, but somewhat informed by comments on MTurk user boards.

In order to provide more information to the workers, we use the the full description of the occupation, including examples of task descriptions from the ISCO-08 documentation. This information often more than doubles the volume of text a worker has to read to properly answer the task. Due to the increase in text to read, the monetary reward for workers was increased.

Neither of the MTurk annotations performed in the second round yielded any significant level of consensus among the workers. By carefully reviewing and annotating the selected occupation descriptions, some causes of ambiguity stands out:

- Some jobs may or may not be performed from home depending on the home. Sewing, wood-working and similar types of craft can plausibly be done from home provided the home is spacious and properly equipped. On a longer time-horizon this may be plausible to many, while few may be able to bring this type of work home on short notice.
- Some jobs can be done from home for a short while, postponing in-person meetings until a later time.
- Some jobs can be done from home, but with a lower quality result. A perhaps poignant example are teachers, who have shown an ability to teach via video even in lower elementary education, but few parents would agree that this is an acceptable long-term or even medium-term solution.
- Some jobs are very dependent on the technology in use at an employer. Filing clerks may have to stay in the office if filing is mostly on paper, but as more and more documents are digital only, such jobs may be done from home. The same is true for certain types of systems administrators in Information Technology: If they are responsible for on-premise data centers they may have to be physically present, while if they are using cloud-computing there are no physical servers to access.
- Some occupations are extremely specialized, and people without experience in those occupations can not be expected to accurately assess WFH feasibility.

This implies that the disagreement in both rounds is not mainly due to human labelling error, but rather represents the possible heterogeneity in evaluations across MTurk workers.^4^ We consider the arithmetic average as a imperfect but good measure. Formally, for occupation *j*, we have *n* different labels. Define

$${\gamma }_{i}\left(j\right)=\left\{\begin{array}{cc}1& if \,\,answer\, \,"Yes"\\ 0.5& if\,\, answer\,\, "Don{^{\prime}}t\,\, know"\\ 0& if\,\, answer\,\, "No"\end{array}\right.$$

and the WFH friendly measure *γ*( *j*) can then simply defined as *γ*( *j*) = ∑ *γ*_*i*_( *j*)*/n*. Note that to assign the value 0.5 to the answer “Don’t know” is somewhat arbitrary, since “Don’t know” may not imply that half part of the jobs in this occupation can be done from home. An alternative is simply to drop these evaluations, which we have also tried and the main results do not change much.

Using the average annotation may work well given the uncertainties outlined above: Annotations are likely to reflect the experience and expectations of the annotator, and aggregating the knowledge of several annotators can provide a more accurate picture of WFH feasibility, reflecting the fact that occupations, employers and employees are not a homogeneous figure. For most of the occupations we revisited, we would be skeptical of any binary label. One may question our practice of using the average value as the remote-friendly measure. The problem is most serious for the “lack of consensus” occupations where at most three respondents that agree with each other. We could, however, treat evaluations of these occupations as missing and assign either all 1 or all 0 to those occupations. This way, we treat jobs of these occupations as either all WFH friendly or all non-WFH friendly, thus establish the lower or upper bound of the prevalence estimate, respectively. Note that no restriction/assumption is made for these occupations when constructing these bounds, this bounding practice is very similar to the so-called “worst case” bounds in the partial identification literature, see for example Manski (2003).

An interesting question is whether the changes made in the second round, namely selecting only MTurk workers with high acceptance rate and presenting more detailed task descriptions, is useful in terms of getting “better” responses. One might consider to perform a two-sample T-test on the hypothesis that the mean responses in round one and round two are the same. However, the T-test requires strong assumption on the underlying distributions. We also cannot appeal to the asymptotic results given the small sample sizes we have. Instead, we apply a permutation test which imposes no distributional assumptions and is valid in small sample (Good 2005). For 76 of 90 occupations that were submitted to MTurk in both rounds, we fail to reject the null hypothesis that the WFH measure from round one and that from round two is the same at 5% level of significance. In other words, for the majority of “uncertain” occupations, the changes we made in the second round did not have significant effects on the results. However, we should note that increasing the sample size will nevertheless improve the estimates’ precision.

Some aspects of our approach are worth further discussion. It is obvious that the workers performing the classifications are not labor market subject matter experts, and so the results are not authoritatively reflecting the intention and original meaning of the creators of ISCO. In addition, respondents to our task on MTurk likely reside in different countries. However, we did not try to correct for possible cultural/technological differences—some jobs that cannot be performed from home in one country may be possible be performed from home in other countries. These are clear limitations of our approach. So in a way, We should consider the evaluations as “international”, which is also true for the ISCO-08 standard itself. However, as shown later in Sect. 5 where we run several consistency checks, these limitations may not affect the reliability of our measure very much.

## Results

Combining our occupational specific WFH measures and the labor statistics per occupation in Norway,^5^ we find that in 2019 around 38 percent of the jobs in Norway can be performed from home.^6^ Applying the bounding approach discussed in Sect. 3, we get the lower bound for this prevalence to be 24% and the upper bound to be 51%. The range is somewhat wide, but still informative.

Splitting into ISCO-08 major occupational groups, we estimate what percentage of jobs in these groups are WFH friendly. The results are presented in Table 1. The share of jobs that can be performed remotely varies from 2 to 66 percent. “Managers”, ‘clerical support workers” and “Professionals” are groups where many of the employees can work from home. Only a small fraction of workers in occupations like “elementary occupation workers” and “plant and machine operators assemblers” can work from home. Table 1 also reports the lower and upper bound for the percentage of WFH friendly jobs in each occupation group.

**Table 1:** Percentage of occupations are WFH-friendly across occupational group

	Percent WFH friendly			
Occupational group	Estimate	Lower bound	Upper bound	No. of jobs
Managers	65.7	40.7	83.8	222,678
Professionals	57.4	40.2	56.4	652,356
Technicians and associate professionals	42.7	24.4	53.6	374,858
Clerical support workers	63.0	57.4	64.9	169,230
Service and sales workers	26.7	7.7	59.9	573,415
Skilled agricultural, forestry and fishery workers	17.0	16.7	18.2	21,631
Craft and related trades workers	12.0	2.0	27.9	219,843
Plant and machine operators and assemblers	7.0	6.6	7.2	163,197
Elementary occupations	1.7	1.7	1.7	134,400
All occupations	38.3	24.0	50.9	2,531,608

### WFH feasibility: job and worker characteristics

**Fig. 2:**
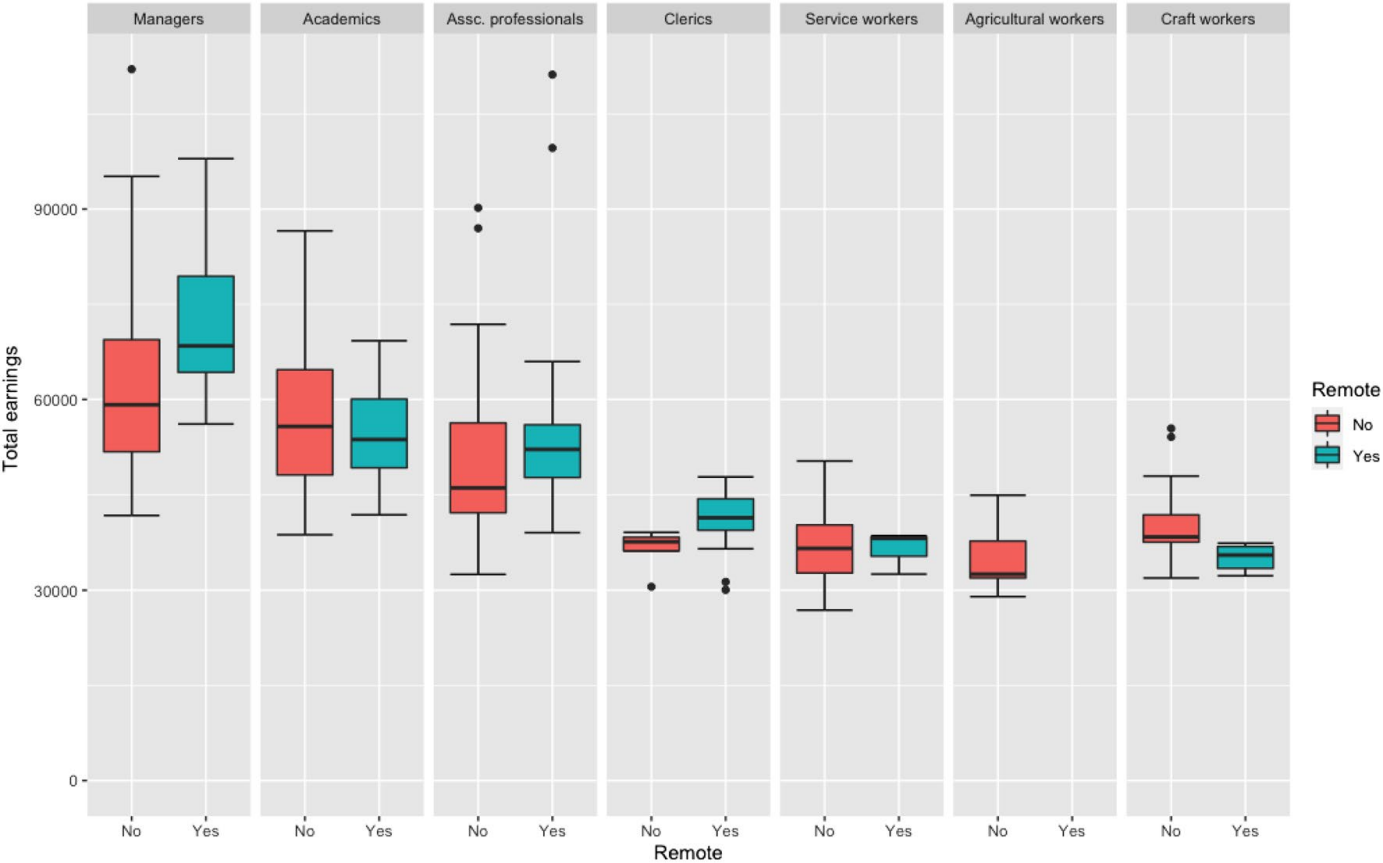
Earnings for WFH and non-WFH friendly jobs across different ISCO groups

We have also access to several administrative registers from SSB, which contains detailed information on workers and their jobs. This enables us to find what types jobs that can be worked from home. Jobs are characterized by wages and working hours. In general, WFH friendly occupations also pay better, as shown in Table 2. The difference between WFH friendly and non-WFH friendly pay is much less pronounced when we split the data by occupational groups, and the pattern is not unequivocal (Fig. 2). In general, there is a wage premium for WFH friendly jobs. However, the difference is minimal for the occupation “professional”. In addition, WFH friendly jobs are more likely to be full-time jobs, indicating that workers with less WFH friendly jobs are also less likely to have a stable job.

**Table 2:** Monthly wage: WFH and Non-WFH jobs

WFH friendly measure	No. of occupations	No. of jobs	Average earnings	Median earnings
High (> 0.8)	53	391,873	55,576	50,792
Medium (0.2—0.8)	202	1,396,139	44,987	43,440
Low (< 0.2)	136	665,653	43,948	42,800

Previous literature shows that workers with different characteristics sort into different occupations. Some groups of workers face more challenges in labor market than others, such as lower-skilled young workers, lone parents and workers with migrant background (Barrett 2010). Unfortunately, our results show that these workers are also less likely to have WFH friendly jobs and thus are impacted disproportionately by the shutdowns and social distancing policies. We find a strong positive correlation between education levels and WFH possibilities. Among those with at least a master’s degree, more than 55% have a WFH friendly job, while only 15% among those with only primary/elementary education have such a job. Older workers are found to be more likely to have a WFH friendly job. Workers over 40 years old have a chance of 40% to be able to WFH, while workers below 30 have only a 30% chance. These observations imply that low-skilled young people are likely be impacted by the crisis particularly hard. Lone parents often work less hours and earn less than others, partially due to the need of caring for dependent children. They do not have similar ability to diversify income shocks and share caring responsibilities of children as couples. This makes them probably the most needed group of WFH. However, our results show that they are also least likely to have WFH friendly jobs among workers of different family statuses.

We also find that workers with a migrant background have much less chance (32%) to have WFH friendly jobs compared with native workers (40%).^7^ However, labor market qualifications and prospects vary widely among these workers. Table 3 shows the percent of workers with WFH friendly jobs by country background. Workers with migrant background from North America and Oceania top the table, while those from Africa are found at the bottom of the rank. This finding is yet another example of the findings that immigrants from low-income countries face disadvantages in many context in the labor markets in developed countries (Careja 2019).

**Table 3:** Percent of Workers with WFH friendly jobs: Country background

World regions	Percent WFH friendly (%)
Native workers	39.5
Workers with migrant backgrounds	32.2
EU28/EEA	33.0
Other European countries outside EU28/EEA	32.3
Africa	26.0
Asia including Turkey	30.1
North America	43.6
South and Central America	32.6
Oceania	42.2

There is also a clear difference across genders. Female workers are more likely to have WFH friendly jobs than male workers, and thus might be less exposed to the social distancing policy. However, there has been argued that the possibility to work from home might actually not be beneficial for female workers as they often have to take on additional housework in this situation (Collins et al. 2020).

To account for possible correlations between these characteristics, we estimate the average marginal effects for these variables on the probability of having a job that can be performed from home (represented by our WFH friendly measure). The patterns remain the same as discussed above. To summing up, our results suggest that those who are already disadvantaged in the labor market, such as workers with a migrant background, young workers and lone parents, are more likely to have non-WFH friendly jobs. Thus, the pandemic and the government’s attempts to mitigate this crisis may have quite an uneven impact on the working population. This hypothesis is consistent with what actually happened during the first weeks of the crisis in Norway: these groups of workers are more likely to be temporally laid off (Alstadsæter et al. 2020). Policies aimed especially towards these particular groups should have a high priority on the government’s list. Although the results are based on Norwegian data, We believe that our findings can be informative for other countries as well, considering that workers of same occupations in different countries often share similar characteristics. In fact, Mongey et al. (2020) studied which workers are more likely to bear the burden of social distancing policy and found similar results as ours. However, while the WFH friendliness is a strong positive predictor of the probability of job losses, institutional differences across countries may play an important role on its magnitude (Adams-Prassl et al. 2020).

### Variation of the prevalence of WFH friendly jobs across different regions and industries

**Fig. 3:**
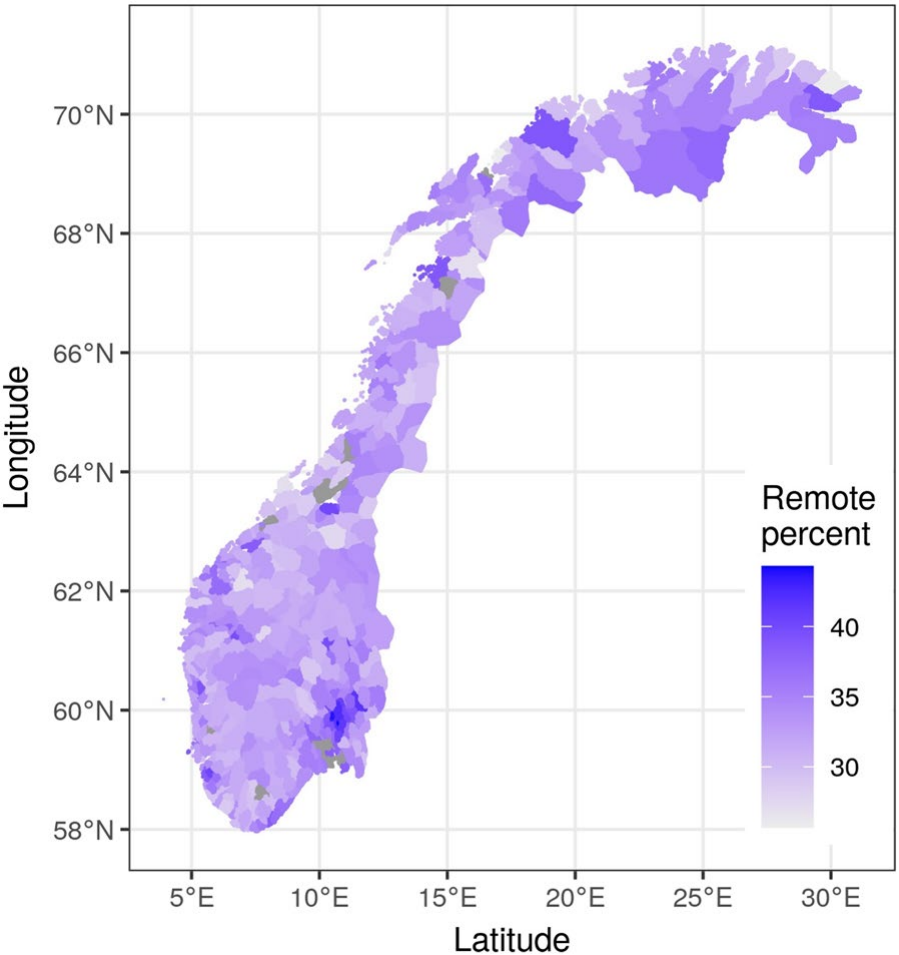
Percentage of workers who can work from home, Norway

The geographic location of jobs has been a point of interest for years, amid both pressure for workers to centralize and specialize, and fears of increased inequality between cities and rural areas. Figure 3 shows the percentage of workers who can work from home in Norway. There is large heterogeneity across different regions. We estimate that over 42 percent of the jobs in Oslo can be done from home. On the other end of the spectrum, in some small rural municipalities in northern Norway just over a quarter of the jobs can be done from home. As we expected, cities have a higher share of WFH friendly jobs, which may be fortunate considering the greater need for social distancing in urban areas. The pattern looks clear, especially in the area surrounding Oslo. Other major cities in Norway like Bergen, Trondheim, and Stavanger stand out on the map as well.

**Fig. 4:**
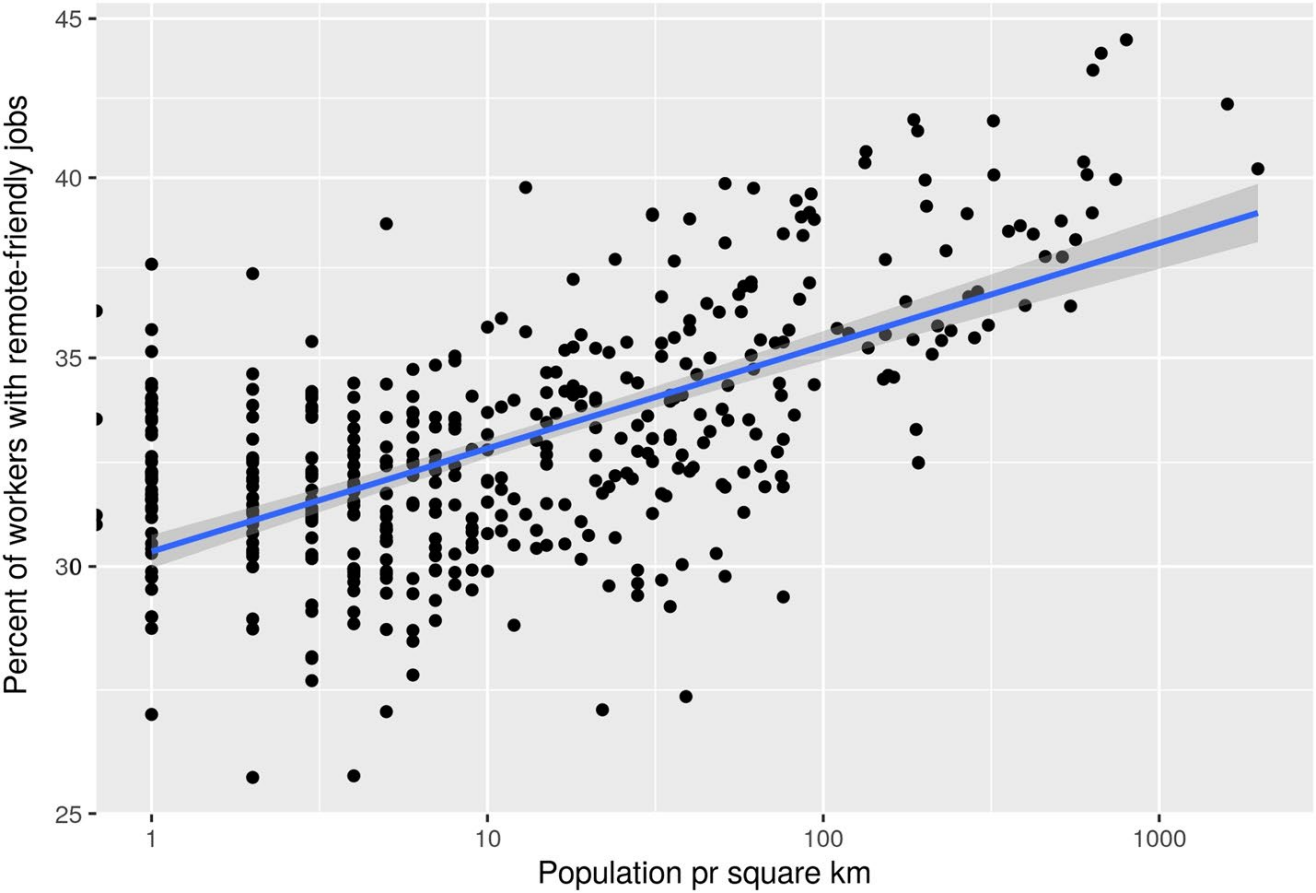
Shares of Remote feasible jobs and population density

By introducing a measure of urbanness, we can analyze the relationship more formally. We use population per square km as a proxy for urbanness. From Fig. 4, we see a clear correlation between “urbanness”, or population pr km^2^, and the prevalence of remote-friendly jobs. Denser populated areas imply greater risks of COVID-19 spread, but this increased risk may be mitigated by better opportunities for remote work.

There is also significant variation in the distribution of WFH friendly jobs across different industries. Industries such as Financial and Insurance (85%), Information and Communication (77%) have the highest share WFH friendly jobs, in contrast to primary and secondary industries which typically have much lower values ranging from 20 to 30%. There are also few jobs that can be worked from home in accommodation and food service (14%), Transportation (25%) and Arts, entertainment and recreation (28%).Holgersen et al. (2020) studied the impact of Covid-19 crisis on the labor demand in Norway using vacancy posting data and found that these industries are among the industries that experienced largest drops in labor demand.

### European results

The European statistical agency, Eurostat, publishes data on employment by ISCO-08 major groups. Combining the results presented in Table 1, we can use these data to estimate the prevalence of WFH friendly jobs in Europe. Note that we have used the Norwegian employment sizes as the weights when aggregating from the ISCO-08 unit groups to the major groups. Since the compositions of occupations can differ from country to country, the “true” weights can differ and lead to potential bias.

**Fig. 5:**
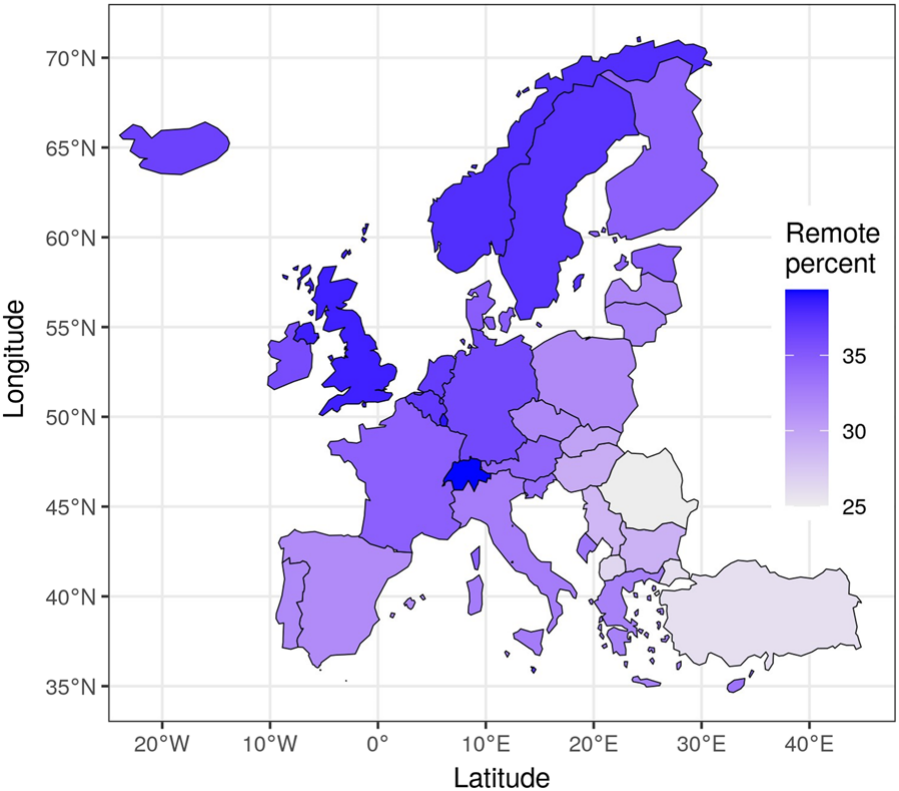
Percentage of workers who can work from home, Europe

Figure 5 presents the geographic variation of predicted share of jobs that can be worked from home across Europe. We observe a considerable difference. The countries with the highest share of WFH friendly jobs are Switzerland, Luxembourg, Norway and Sweden. On the other end, the countries with the lowest share of WFH friendly jobs are mostly less developed countries in southeast Europe, such as Turkey and Romania. As Dingel and Neiman (2020) suggested, there seems to be a clear positive relationship between GDP per capita level and predicted share of WFH friendly jobs. Interesting, the pattern on the prevalence of WFH friendly jobs we find above is very similar to the actual observed incidence of telework in Europe reported by Gschwind and Vargas (2019). They find also “a rough North/South and East/West divide in the incidence of telework”.

## Validation and consistency check

### Consistency check: using alternative classification results

There are several very recent analyses that study the prevalence of WFH jobs for different countries: Dingel and Neiman (2020), Brynjolfsson et al. (2020), Mongey et al. (2020), and Hensvik et al. (2020) for the United States, Alipour et al. (2020) for Germany, and Barbieri et al. (2020) for Italy. Unlike our study, they rely on different surveys, and the results are established on their national occupation classifications.

**Fig. 6:**
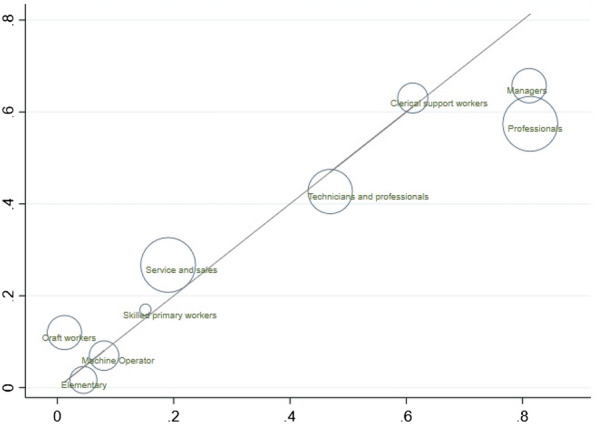
The US classification and ours, ISCO major groups

Although their results are based on the OES/SOL occupation groups, Dingel and Neiman (2020) manage to use the crosswalk between the OES groups and the ISCO-08 groups. This crosswalk provides an opportunity to compare our results with theirs. As a robustness check, we have redone the above analyses using the US classification results. Given the many to many nature of the crosswalk, we do not expect that their results and ours agree with each other on the lower levels of occupation groups, but they should be similar on a more aggregated level, such as the ISCO major group. Using the US classifications, the overall share of remote-friendly jobs in Norway is estimated to be around 43%, slightly higher than our estimate 38%. Figure 6 presents the scatter plot of shares of jobs predicted using their measures against those using ours for 9 major ISCO occupation groups together with the 45-degree line. The bubble size represents the employment numbers in Norway. There is a strong positive correlation between these results. The key patterns we found in Sect. 4 on earnings, worker’s characteristics, geographic and industry variations remain to be the same when using the results by Dingel and Neiman (2020).

### Consistency check: comparing with actual observed incidence of WFH

There was almost no surveys that particularly focus on the topic of home working/ “telework” in Norway. The only exception is a recent survey by the Norwegian Institute of Transport Economics (TØI) that is designed especially for the COVID-19 situation. The main focus is the effectiveness of working from home (Nordbakke 2020). In the survey, 950 respondents are asked about their working situation for a given day, 19th March, 2020. Among them, 32 already work from home already before the Covid-19 crisis. 67 have the possibility to work from home, but still go to work on that day. And 401 have the possibility and actually work from home on that day. Summing up, we obtain an overall estimate of the prevalence of WFH friendly jobs (52%). This estimate is considerably higher than our estimate. However, the survey is based on the job situation only for a given day and can potentially lead to a upward bias. It is unfortunate that the survey contains very limited information: no characteristics of jobs or workers are collected, which makes further comparison of our results impossible.

Another survey that contains questions concerning possibilities of working from home is the Norwegian labor force survey in 2017. The question was whether the respondent had the *opportunity* to work from home when she/he wants to. This is not to say that the job could be performed remotely in its entirety, and neither to say that those who weren’t given the opportunity all have jobs that cannot be performed from a home office. The results are discussed by Nergaard et al. (2018). According to the labor force survey, around 35% have the possibility to work from home, which is very close to what we find in the current analysis. However, as discussed earlier, the magnitudes of these two estimates may not be directly comparable. What is really interesting to check is the distribution of WFH jobs across different worker, occupation and industrial groups. Consistent with our results, the survey also finds that the share of workers who can work from home increases with worker’s education levels. The results on occupation and industry groups are broadly similar to what we obtained but with some differences. For example, around 29 percent of workers in “Clerical support workers” who participated the survey responded that they had the opportunity to work from home at times, much lower than the results we obtained. This is likely attributable to the distinction mentioned above: Not having the *opportunity* to work from home does not necessarily mean that the job can not be performed from home.

### Validating results against job-ads

Another way to evaluate the results from Mechanical Turk is to use advertisements from the Norwegian welfare administration (NAV). These job advertisements have been published as open data by NAV, and contain the text, title, employer information, and annotations made by subject matter experts at NAV including the occupational code of the job. Because the possibility to work from home is a perk for many, some employers mention it in their job ads to attract candidates. We search the texts for mentions of “hjemmekontor” and “heimekontor”, two distinctive words unlikely to mean anything other than the possibility of working from home. We find that there are quite few, only around 2.5 among every 1000, announcements that actually include these words. Obviously, far from all announcements of jobs that can be performed remotely include these words. We cannot derive the total number of WFH friendly jobs from these job ads data alone. However, it says something important about the relative frequency of WFH-friendly jobs across the occupational groups.

Table 4 presents the actual observed relative frequencies from the job announcement data and those predicted using our WFH friendly measure. Large discrepancies are found for three major groups “Professional”, “Technicians and associate professionals” and “Clerical support workers”. Our measure from the MTurk predicts more cases of the mentions of “home office” than what are actually observed in the NAV data for the first group, and less cases for the last two groups. It could be that our MTurk results are biased. However, we think it is more likely that employers in these occupations have different perceptions on the importance of the “working from home” feature to attract potential candidates. Note that in the job announcements, exact wage is seldom listed. From Fig. 2, we see that for the last two groups “Technicians and associate professionals” and “Clerical support workers”, wages of WFH friendly jobs are on average much higher than wages of non-WFH jobs. To some extent, being able to work from home can be seen as proxy of high wages in this two occupations. So the employers may have stronger incentives to include these words to attract potential applicants. Overall, we think the correlation we see in Table 4 is decent considering the spuriousness of the data.

**Table 4:** Relative frequency of WFH possibilities across ISCO groups

	Relative WFH frequency		
Occupational group	MTurk (%)	Job ads (%)	Difference (%)
Managers	3. 9	3. 1	0. 8
Professionals	67. 6	48. 0	19. 6
Technicians and associate professionals	13. 3	27. 2	− 13.9
Clerical support workers	3. 5	9. 9	− 6.4
Service and sales workers	5. 8	9. 1	3. 3
Skilled agricultural. forestry and fishery workers	0. 0	0. 0	0. 0
Craft and related trades workers	5. 2	1. 2	4. 0
Plant and machine operators and assemblers	0. 5	1. 4	1. 0
Elementary Occupations	0. 2	0.1	0. 2

## Conclusion

In this paper, we propose a new method to evaluate the prevalence of WFH friendly jobs in an economy. In particular, we ask respondents from MTurk to evaluate whether the main tasks of occupations can be performed from home and establish a measure of the feasibility of WFH for each occupation. Compared with transitional approaches via experts or surveys, our approach is easier to implement, costs less and takes shorter time.

The fact that the WFH feasibility is evaluated on the occupation level but not on the job level may lead to potential bias since it essentially ignored the heterogeneity across jobs within the same occupation. A related issue is that it might be difficult to assign a binary label to some occupations. So far we have treated the lack of consensus among the respondents as an indication of certain occupations being “problematic” and try to cope with this problem by aggregating the answers. However, in future practice, we may consider allowing for more detailed labels or asking the respondents to provide own estimate of likelihood on a given scale directly. Another concern is that the respondents from MTurk are not labor market subject matter experts and reside in different countries. These could also lead to bias since the respondents are subject to possible cultural and technological differences and might misinterpret the task descriptions.

These concerns highlight the need to check the reliability of our method. To do this, we have performed several validation tests. We compare our results with the classification results of Dingel and Neiman (2020), with the WFH incidence derived from two surveys in Norway (Nordbakke 2020; Nergaard et al. 2018) and finally with the results generated from the job advertisements in Norway. We do not find evidence of large bias in our estimates. Although none of these checks can be considered as formal tests of reliability, the positive results enhanced our belief that our approach is a suitable alternative to the mainstream methods.

This analysis is an attempt to combine conventional (the administrative register and official statistics) and unconventional (data from a web-based crowd-sourcing platform) sources for statistical and research purposes. The results we have found suggest that alternative approaches to collecting such information are feasible.

## Data Availability

This paper uses both publicly available data and restricted data. The public available data and code for replication can be found at https://github.com/radbrt/working from home. The restricted data that contains information on individual workers are available from Statistics Norway but restrictions apply to the availability of these data, which were used under license for the current study, and so are not publicly available. Data are however available from the authors upon reasonable request and with permission of Statistics Norway.

## Abbreviations

ISCO: International Standard Classification of Occupations
MTurk: Amazon Mechanical Turk
NAV: The Norwegian Welfare Administration
SSB: Statistics Norway
TØI: The Norwegian Institute of Transport Economics
WFH: Working From Home

## Footnotes

1 We will refer to workers with WFH friendly jobs and non-WFH jobs as WFH workers and non-WFH workers respectively hereafter.

2 We thank an anonymous referee for pointing out this potential source of bias.

3 Note that we haven’t include 9 “residual” occupations in the second round, since the descriptions of tasks are often not quite informative. For example, occupation 1219 is defined as “This unit group covers business services and administration managers not classified elsewhere…”.

4 We consider most of the lack of consensus to stem from above mentioned factors, but noise is still a contributing factor: One may ponder how in all three rounds of MTurk annotation at least one worker submitted that a prison guard could work from home.

5 Statbank, Statistics Norway. Data can be obtained at https://www.ssb.no/en/statbank/table/12542/.

6 To be precise, this should be stated as “ the average of our occupational specific WFH friendly measures for all registered jobs in Norway in year 2019 is 0.38”. Given that our measure of WFH friendliness is an estimate of the mean of a binary variable, we can interpret our measure as the probability of a job of the given occupation can be worked from home. We should keep in mind that this simplification is obviously based on strong assumptions.

7 By workers with a migrant background, we refer to both immigrants and Norwegian-born to immigrant parents. If we limit to only immigrants, the chance is even lower, 26%.
